# Recent Advances in Multi-Functional Coatings for Soft Magnetic Composites

**DOI:** 10.3390/ma14226844

**Published:** 2021-11-12

**Authors:** Emir Pošković, Fausto Franchini, Luca Ferraris, Elisa Fracchia, Jana Bidulska, Federico Carosio, Robert Bidulsky, Marco Actis Grande

**Affiliations:** 1 Department of Energy (DENERG), Politecnico di Torino, Viale T. Michel 5, 15121 Alessandria, Italy; fausto.franchini@polito.it (F.F.); luca.ferraris@polito.it (L.F.); 2 Department of Applied Science and Technology (DISAT), Politecnico di Torino, Viale T. Michel 5, 15121 Alessandria, Italy; elisa.fracchia@polito.it (E.F.); federico.carosio@polito.it (F.C.); marco.actis@polito.it (M.A.G.); 3 Consorzio INSTM, Via G. Giusti 9, 50121 Florence, Italy; 4 EPMA PM R&D Centre, Faculty of Materials, Metallurgy and Recycling, Technical University of Kosice, Park Komenskeho 10, 040 01 Kosice, Slovakia; jana.bidulska@tuke.sk; 5 Asian Innovation Hub, Budulov 174, 045 01 Moldava nad Bodvou, Slovakia; robert.bidulsky@asihub.org

**Keywords:** powder metallurgy, Soft Magnetic Composite (SMC), organic and inorganic layers, metal oxides, resins, iron losses, magnetic permeability, mechanical strength, materials for electrical machines and RF devices

## Abstract

During the past 50 years, the aim to reduce the eddy current losses in magnetic cores to a minimum led to the formulation of new materials starting from electrically insulated iron powders, today called Soft Magnetic Composites (SMC). Nowadays, this promising branch of materials is still held back by the mandatory tradeoff between energetic, electrical, magnetic, and mechanical performances. In most cases, the research activity focuses on the deposition of an insulating/binding layer, being one of the critical points in optimizing the final composite. This insulation usually is achieved by either inorganic or organic layer constituents. The main difference is the temperature limit since most inorganic materials typically withstand higher treatment temperatures. As a result, the literature shows many materials and process approaches, each one designed to meet a specific application. The present work summarizes the recent advances in state of the art, analyzing the relationship among material compositions and magnetic and mechanical properties. Each coating shows its own processing sets, which vary from simple mechanical mixing to advanced chemical methods to metallurgical treatments. From state of the art, Aluminum coatings are characterized by higher current losses and low mechanical properties. In contrast, higher mechanical properties are obtained by adopting Silicon coatings. The phosphates coatings show the best-balanced overall properties. Each coating type was thoroughly investigated and then compared with the literature background highlighting. The present paper thus represents a critical overview of the topic that could serve as a starting point for the design and development of new and high-performing coating solutions for SMCs. However, global research activity continuously refines the recipes, introducing new layer materials. The following steps and advances will determine whetherthese materials breakthrough in the market.

## 1. Introduction

Soft Magnetic Composites (SMC) derive from the strong interaction between different but adjacent industrial environments. During the past three decades, powder metallurgy offered an innovative branch of raw materials and products that constituted a starting point for a brand new concept of ferromagnetic materials, with many advantages in the electrical machines [1,2,3,4,5], signal and power electronics, and EMI filtering sectors [6,7,8]. During recent years, technology underwent a rapid development, with significant differentiation of processes and materials. While the first documented attempts of obtaining an iron core from compacted powder date in the late 1960s, the actual SMC development started in the middle 1980s [9,10].

Compared to that of laminated steels, soft Magnetic Composites SMC represents a significant step in reducing the eddy current losses [11]. SMC structure consists of electrically insulated iron powder grains, which bring the lamination concept to an extreme and exploit in magnetically isotropic material. In most cases, the insulating material acts as a binder for the metal powder. Therefore, it can be the most critical part of the SMCs, playing a dominant role in the research activity.

The coating process is crucial; each coating system may be prepared by different techniques. Among the various coating technologies, some are largely diffuse in literature; for instance, the nanocomposite coatings [12]. These coatings are a combination of nano-compounds, polymer matrix, additives, and solvents adopted for producing multifunctional coatings. Other coatings such as bio-based polyurethane (PU) have a wide range of applications, from thermally stable coatings to corrosion resistance coatings [13].

On the other hand, other coatings processes as Plasma Electrolytic Oxidation Process (PEO) are adopted, for example, to realize corrosion-resistant adhesive coatings. The PEO coating seal may be realized by hybrid sol-gel coatings, mineral sealing layers, or electroless nickel plating [14]. Even cold gas dynamic spraying may be taken into account as a possible coating process. In this sense, Temitope et al. largely studied cold spraying techniques [15].

The volume ratio between the magnetic powder and the insulating/binding material is, in some way, proportional to the final magnetic and energetic properties of the SMC. Therefore, it has to be maximized, reducing the insulating material to only a very thin layer around each single metal particle [16,17].

On the contrary, the layer thickness is inversely proportional to the layer’s binding effect and, again, inversely proportional to the macroscopic electrical insulation. The research activities mainly focus on optimizing thinner, uniform binder layers, which also show good mechanical and dielectric properties.

Because the SMCs still represent a tradeoff between mechanical, electrical, and magnetic aspects [18], choosing which property has to be held first for the specific final application is crucial.

The granulometry of the powders [19,20] and the number of pores [21] in the final component are critical parameters in the SMC mixes. As for the latter, it is well known [22,23,24] that pores act as crack initiators. Due to their presence, the stress distribution is inhomogeneous across the cross-section and reduces the effective load-bearing area. Plastic deformation during the pressing pressure results in higher iron losses. Porosity harms the magnetic properties such as magnetic induction and permeability as well the coercive force. As powder sizes regard, the biggest particles begin conducting a relevant amount of eddy currents at higher frequencies, reducing the insulation effectiveness. Furthermore, the particle size is inversely proportional to the inner demagnetizing field, which causes most of the hysteresis losses in the SMCs. Smaller particles generally show lower eddy current losses, together with higher hysteresis losses and earlier saturation, meaning that an increase in the external magnetic field does not provoke a further rise in the magnetic induction. The final heat treatment is the last key point in the SMC process and represents the third tradeoff. Heat treatment at a high temperature can be very useful in raising the mechanical properties and positively recovering the metal’s internal stresses, with benefits on the hysteresis cycle. Inversely, a too high temperature easily degrades or destroys the electrical insulation, raising the eddy current losses quickly.

The research approach to the aforementioned issues may be manifold, resulting in various processes and materials. The change of properties is most evident between materials having a different field of applications. For example, radio-frequency materials, such as the EMI absorbers, are made of finer powders, well-insulated through a relatively thick insulating layer, while an SMC used in an electrical machine shows thicker, late saturating powder grains with good large-cycle behavior and the thinnest coating layer. The selection of materials for the insulating layer plays the main role in the properties and applications of SMCs. In general, the insulating layer can be organic or inorganic, but sometimes hybrid solutions are adopted. The inorganic layer is designed to be arranged at high temperatures to provide mechanical strength and recovery hysteresis losses (over 400 °C), usually increasing after the manufacturing processes (grinding, compaction, and so on). On the other hand, some of these coatings are characterized by long process steps, resulting in low cost-efficiency.

The organic coatings are characterized by good electrical insulation, thanks to their high capability to completely cover the ferromagnetic particles. The processes come from polymer technologies: compression molding, curing thermosetting materials, and injection molding for thermoplastic materials. These techniques allow a theoretical higher production due to the high cycle time. The major drawback of the organic coatings is the heat treatment, limited to around 400–500 °C in the case of silicon-based resins.

The layer choice depends on the final application for the material. The frequency range, mechanical aspects, thermal stability, and magnetic permeability behavior, are totally different in electrical machines or EMI filtering sectors. In this respect, it seems evident the need to uniform the protocol of materials investigations. In the case of SMCs applied to electrical machines, the iron losses performances, the B-H curve, which is magnetic induction B (in [T]) as the function of the magnetic field H (in [A/m] or [Oe]) is required, as well as its mechanical resistance [25]. For these reasons, the units should be uniform and comparable to gain a comprehensive understanding of the involved parameters and evaluate the layer performances correctly. Therefore, only SI units will be considered to compare different typologies of SMCs coating systems.

This review manuscript aims to compare magnetic, energetic and mechanical performances that will be discussed for each type of layer, which will be grouped as a function of the nature of the coating. The behavior of different inorganic and organic layers will be reported in the following paragraphs: the latter will be further subdivided into resins and thermoplastic polymers.

## 2. Purpose Descriptions

This work aims to summarize the various coating systems adopted in SMCs. Both inorganic and organic systems were considered and, for each category, the available properties reported in the literature were compared. Coatings are compared in terms of magnetic properties, iron losses and mechanical properties (when available). In addition, the comparison will focus on the magnetic permeability and transverse rupture strength (TRS): in this sense, graphical comparisons were made considering the literature data.

Literature properties will be compared to stress the relation between maximum permeability, transverse rupture strength (TRS) and energetic properties. Specific bubble charts were made to compare these properties. The production processes are grouped for their typologies (similar colour tone mean similar process methods), and the bubble chart is arranged to show the value of the specific iron losses, where the small ones have the better energetic performance (lower iron losses). Furthermore, each bubble was labelled with the iron losses values (in [W/kg]) at 1 T@50 Hz.

## 3. Inorganic Coating Systems

This section reports the inorganic coating systems commonly adopted in SMCs. Mainly, silica, silicon, phosphates, ferrites, aluminum alloys, alumina, and titanium systems were shown, as well as their production processes. Bubble charts highlight the properties found for each type of coating.

### 3.1. Silica

SiO_2_ can be used as an inorganic coating for soft magnetic composites, especially as amorphous silica. Silica gel and aerogels are made of silica too, and they can be used to improve the coating process [26,27,28,29]. In addition, silica is already adopted in different industrial sectors as an electrically insulating material and also presents important characteristics related to microstructure and thermal stability. For these reasons, it can be used as an inorganic coating to cover the magnetic powder by means of nanocomposite sized particles or as silica coating compound together with phosphates and other oxides for ferromagnetic materials [28,30,31,32]. Moreover, the coating system based on SiO_2_ represents a solution to the problem caused by organic layers, which decompose at high temperatures, making annealing and heat treatments very limited and impossible at temperatures over 500 °C [33,34,35,36]. It also ensures high resistivity and limitation of conducting paths, reducing eddy currents, confining them inside the particles and obtaining limited core losses [28,33,37,38].

According to Yang et al. [38], amorphous SiO_2_ layers fabricated by controlled hydrolyzation show specific core losses around 3.5 W/kg, for the operating frequency 50 Hz and the magnetic induction equal to 1 T (which represents a typical value for magnetic materials used in electrical machines). The study, however, does not report the value of magnetic permeability at low frequencies. Wu et al. [37] obtained shallow iron losses for low and medium operating frequency values using the fluidized chemical vapor deposition process (FCVD) combined with the subsequent spark plasma sintering (SPS). The specific iron losses at 1 T@50 Hz are 1.5 W/kg. The maximum mechanical value of 96.76 MPa was obtained by Strečková et al. [27], with the adoption of SiO_2_ nano-rods, which are chemically incorporated into the phenolic polymer matrix through the modified sol-gel method. However, the mechanical strength was not reported. On the other hand, the expected mechanical properties are generally low [30]. Pang et al. [29] showed a flexural strength of 72 MPa using silica through the aqueous-based sol-gel process.

The drawback of silica-coated SMCs is their brittleness, limiting the material compressibility, density, magnetic phase content, and permeability. Moreover, iron losses are related to the SiO_2_ content percentage in weight. The limit percentages are about 1.25 wt.%, as shown by Liu et al. [39].

The hysteresis losses coefficient is lower for Fe-SiO_2_ SMCs due to the ability of the coating to operate at high annealing temperatures; the coefficient decreases as temperature increases. Differently, the eddy current loss coefficient increases with higher annealing temperatures. This behavior occurs since annealing causes a reduction of particles distortion, thus a decrease in the electrical resistivity. Moreover, other positive effects due to the increase of the annealing temperature are related to the increase of initial and maximum permeability, the increase of magnetic induction, and the reduction of the coercivity. Summing up the two factors’ contributions, the total core losses in the SiO_2_ coated SMCs can be controlled, obtaining promising results.

#### Production Methods of Silica-Coated SMCs

Many coating processes are primarily based on the Fe-Si spherical ferromagnetic particles (gas-atomized Fe-6.5 wt.% Si powder) [27,28,33,36,37,39] or sometimes irregular powder shape [29,30,38]. For this reason, it is important at first to define the typology and sizes of iron powder. The production of the coating can be based on chemical or mechanical methods. Furthermore, also as part of the coating system, the CIP (fine carbonyl iron powder) combined with RIP (coarse reduced iron powder) can be used, exploiting their small size particles, respectively 2 µm compared to 100 µm of RIP [34]. In this case, the fine carbonyl iron powder helps the electric and energetic properties in the high-frequency range.

SiO_2_ coated SMCs can be produced by reverse microemulsion [40]. This method needs tetraethoxysilane (TEOS) and allows the production of very thermal stable materials. On the other hand, the complete process takes a long time.

All the studies started with iron powder or iron-based powder, following a process similar to the previous one in which ammonia and complex organic compounds were mixed and, after many hours of stirring, washed and dried. Other similar procedures were followed in more recent researches [31,34,35], coupling ammonia and complex organic compounds with the Stöber method [41] and spark plasma sintering (SPS). The adoption of the Stöber method allows to separate the magnetic phases effectively by a homogeneous and uniform intergranular insulating phase. The process via chemical vapor deposition (CVD) shows very promising results, as mentioned above [37], and similar techniques are used by Liu et al. [39]. Luo et al. [32] adopted water oxidation combined with spark plasma sintering (SPS): the process is more cost-effective and straightforward than other reported methods.

The mechanical coating process was proposed by Wu et al. [36], adopting ball milling. The compaction and heat treatments can be very different and depend on the adopted method. According to Wu et al. [40], a 500 MPa pressing level was performed, followed by an annealing process in nitrogen at 300, 500 and 600 °C, depending on the specimen, to observe annealing temperature influence. The compaction pressure was only 30 MPa in the case of utilization of SPS. Such production paths require a temperature of around 1000 °C. Other parameters may influence the magnetic behavior, such as TEOS amount and, in general, the entity of all the chemical agents. They have a crucial role in the percentage of silica adopted to cover the ferromagnetic particles, as described by Teixeira de Mendonça et al. [31].

**Figure 1:**
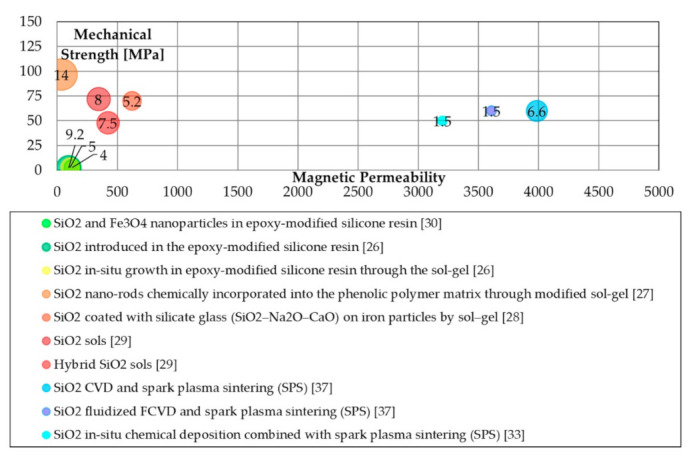
Magnetic (maximum permeability), mechanical (transverse rupture strength (TRS) [MPa]), and energetic properties (total iron losses 1 T@50 Hz [W/kg]) of SiO_2_ coating processes.

In Figure 1, the magnetic, mechanical, and energetic properties were reported based on the applied SiO_2_ coating processes. The methods based on CVD followed by spark plasma sintering show promising results as demonstrated by the relatively high magnetic permeability. Unfortunately, although the achieved values are certainly of interest for a practical application as they would allow for improved energy densities, mechanical performances remain quite limited for all the analyzed processes.

### 3.2. Silicon

In addition to silica, also Si can be used for inorganic layers in SMCs coating due to its good insulating properties. The use of Fe-Si powders in Pressed and Sintered components is studied in [42,43]. Gas atomized spherical Fe-Si powders, showing good electrical resistivity and high ease for coating, were also used in additive manufacturing [44].

Furthermore, as occurring for silica, pure iron powders are also used as the base ferromagnetic particles on which to put the silicon. Given its brittleness, the maximum addition of silicon rarely exceeds 6–7 wt.%.

The maximum permeability obtained at low frequencies can reach over 20,000; for instance, in the case of magnetic materials prepared by Selective Laser Melting (SLM), the magnetic permeability is 24,000 [45]. The total iron losses turned out 2.2 W/kg for 1 T @50 Hz [44]. The reported information seems very promising due to thermal treatment at 1000–1200 °C (sintering in vacuum or Argon). Still, the eddy current losses increase with the increase of the frequency or magnetic induction. This effect is deepened in the work of Tiismus et al. [46], where the total iron losses for 1 T @50 Hz are 8.17 W/kg, whereas, for 1.5T @50 Hz, the value is 83.7 W/kg. Likewise, Goll et al. [47] report losses in medium frequency equal to 6.5 W/kg for 1 T @50 Hz and 95.5 W/kg for 1 T @200Hz.

#### Production Methods of Silicon-Coated SMCs

The production process may vary as a function of the technology: chemical [48], mechanical surface modification [49,50], and recently also additive manufacturing [44,45,46,47].

The chemical method is characterized by magnetic values lower than those deriving from roll [49] and ball milling [50]. Schäfter et al. [49] reported a compaction and sintering at 1100 °C, followed by a new press step and heat treatment at 600 °C. As for the additive manufacturing methods, SLM and Binder Jetting Technology (BJT) were used to produce electrical machines and components [44].

Goll et al. [47] proposed forming the slits in the cross-section perpendicular to the direction of the magnetic flux. In this way, the eddy currents are reduced from 36 W/kg (1 T @50 Hz, no slits) to 23 W/kg (slits with a depth of 2 mm). A further method is based on spherical Fe-6.5 wt.% Si powder consists in the preparation of ribbons through the melt-spinning technique [51]. Each ribbon is subsequently coated with MgO powder, which provides the insulation and adhesion properties to the final piece. The magnetic and energetic properties are promising, while the material’s brittleness gives limitations.

**Figure 2:**
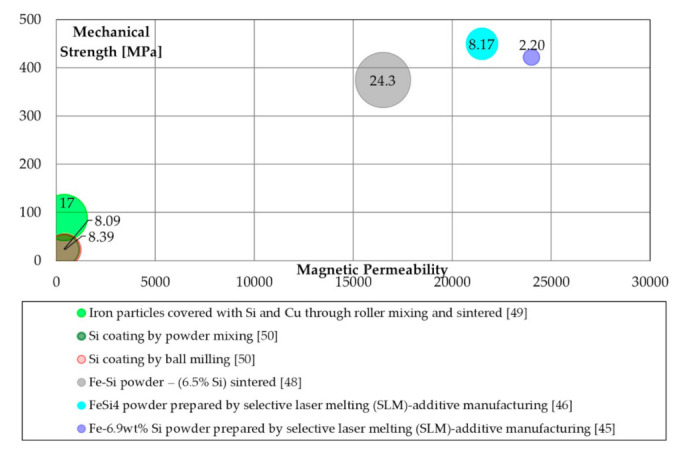
Magnetic (maximum permeability), mechanical (TRS [MPa]), and energetic properties (total iron losses 1 T@50 Hz [W/kg]) of Si coating processes.

In Figure 2, the magnetic, mechanical, and energetic properties of Si coated SMCs are summarized. The bubble chart also includes the sintered silicon iron powder as a reference for magnetic materials based on additive manufacturing. It is possible to note interesting results for the specimens produced by additive manufacturing, but it is crucial to remember their tendency to have very high eddy current losses at medium- and high-frequencies. The iron losses reported in Figure 2 are at 50 Hz and do not show all the energetic behavior of the analyzed soft magnetic composite materials, especially in the high-frequency range.

### 3.3. Phosphates

Phosphating is a well-established method effective in producing thin insulating layers. Generally, phosphate layers reduce the eddy current losses, increase the resistivity, and resist to temperature ranges up to 500–600 °C. Sometimes Cr is added to the ferromagnetic powder to enhance corrosion resistance [52]. In other cases, phosphating the iron surface brings to the Mn phosphate layer, which increases the abrasion and thermal resistance [53], or to Sr and Y amorphous layers with high resistivity [54]. The versatility of phosphates allows them to operate in different conditions. For this reason, coupled with the low iron losses and the cost-efficiency, it represents the reference SMC coating system.

On the other hand, mechanical properties [55,56] and the limitations to the temperature of thermal treatment (usually not over 650 °C) [53,54,57] restrict the large-scale utilization. The characteristics of phosphate coated SMCs are reported in [58,59,60]. Iron loss values are around 5 W/kg (1 T @50 Hz), and the maximum magnetic permeability is about 500. TRS are higher than 40 MPa [61]. These results are related to heat treatment at 500 °C in air, while for other heat processes, as in steam at 530 °C or nitrogen at 650 °C, it is possible to reach TRS of 125 MPa and a maximum magnetic permeability of 850 [61,62]. These results are obtained using warm compaction.

#### Production Methods of Phosphate Coated SMCs

The wet chemistry method is the main phosphate coating process based on orthophosphoric acid H_3_PO_4_ [52,53,55,57,63,64]. A similar approach is performed using zinc phosphate solution [52]. In some cases, the adoption of other elements can improve specific material properties. A particular procedure is described by Tajima et al. [54], involving a simple coating of iron powders with an amorphous phosphate insulator containing various cations: Mg^2+^, Y^3+^, Sr^2+^. Warm compaction (at 150 °C) with die wall lubrication and a pressure of 1176 MPa was used, followed by final annealing in a temperature range between 400 and 600 °C. TRS of approximately 200 MPa was obtained; however, this process is hardly replicable in an industrial environment due to die wall lubrication, the high compacting pressure, and the warm die being over 100 °C.

Xia et al. [52] started their experimental test from FeSiCr powders with an average size of 16.7 µm. The powders were phosphated in two ways: with phosphoric acid H_3_PO_4_ and zinc phosphate Zn_3_(PO_4_)_2_. They both underwent compaction in rigid dies at 1200 MPa and annealing in argon at 200 °C and 500 °C for 1 h. The main difference between the two is represented by the thicker insulating layer of the process based on zinc phosphate solution due to the higher presence of phosphorus. Therefore, Zn phosphated samples show a much lower permeability if compared to that of phosphoric acid samples.

In the work of Liu et al. [65], a phosphate insulation coating was applied on FeSiAl powders (particle size <75 μm). The insulating process firstly adopted the phosphoric acid solution containing phosphoric acid and boric acid. Phenol-formaldehyde resin and alcohol were then added to the final mixture. Magnetic performances for high-frequency applications were evaluated, but the very high compacting pressure (1822 MPa) limits any industrial application. Another method was shown by Chen et al. [66] starting from gas atomized FeSiAl powder, mixed with an aqueous solution of phosphoric acid and using ultrasounds process. The coated powder was then pressed at 2,000 MPa and annealed at 350 °C under a nitrogen atmosphere. During this process, Fe and Al react with the solution under ultrasonic effect. The diffusion of iron and aluminum takes place, in the form of Fe^2+^ and Al^3+^, towards OH^-^. Various phosphates are formed, not providing however high magnetic performances. In the work of Lee et al. [53], pure iron powder (purity 99.99%) is coated through manganese nitrate [Mn(NO_3_)_2_] and citric acid (C_6_H_8_O_7_). The system was then compacted at 800 MPa and annealed and 600 °C in argon to remove residual stresses. The increase of Mn nitride content decreases iron losses, particularly in terms of the eddy current component, while hysteresis was not particularly affected.

Eddy currents dependence on high resistivity is due to the addition of manganese nitrate, which shows good heat resistance. The addition of citric acid makes the coating uniform but limits the iron losses values, eddy currents and hysteresis losses.

**Figure 3:**
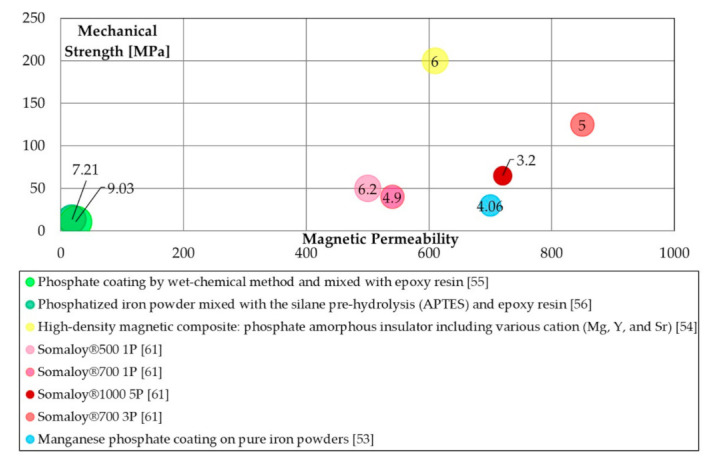
Magnetic (maximum permeability), mechanical (TRS [MPa]), and energetic properties (total iron losses 1 T@50 Hz [W/kg]) of phosphate coating processes.

Figure 3 the magnetic, mechanical, and energetic properties of phosphate coated SMCs. The best mechanical results are obtained with a manganese phosphate coating system, while typical values are around 50 MPa, except for high-density magnetic composites produced with various cations, where mechanical strength reached almost 200 MPa.

### 3.4. Ferrites

Ferrite-based coating layers represent an interesting possibility for SMCs thanks to their thermal resistance and their high electrical insulation and magnetic behaviors. Different ferrites can be adopted for the coating process: Ni-Zn ferrite, Ni ferrite, Mn-Zn ferrite, Co ferrite, et cetera [67]. The more common iron oxides, such as hematite Fe_2_O_3_ [68,69], magnetite Fe_3_O_4_ [70,71,72,73,74], hematite α-Fe_2_O_3_ [75], and maghemite γ-Fe_2_O_3_ [74,76], are obtained in situ and/or added in the mixture to obtain the inorganic SMC layer. However, in some cases, their antiferromagnetic behavior can considerably decrease the permeability [71].

To reach very high frequencies, soft ferrites and ceramic materials may be used; for example, Ni-Zn ferrites reach up to 3 GHz. In any case, for EMI filter applications, it is an excellent solution to cover the ferromagnetic materials and operate in the high frequency. Ferrites in the form Fe_x_O_y_ have more uniform insulating layers than Mn-Zn and Ni-Zn ferrites [77,78,79,80,81,82]. These layers provide thermal stability, insulation, and ferromagnetic behaviors [83]. The joint presence of Fe and Fe oxides in a single component ensures high magnetic permeability and sufficient low iron losses [68]. The main drawbacks are the shrinking during heat treatments, metal-to-metal contact yielding and cracks formation due to their brittleness. To overcome these limitations, different processing techniques are adopted, involving different materials, even including organic resins. Accurate control on particle size is also conducted up to the latest development of nano-sized fibers or hybrid organic-inorganic coatings [84].

Low iron losses, 6 W/kg (1 T @50 Hz), were obtained in the case of composite based on Fe_3_O_4_, which were firstly prepared by oxidizing under appropriate controllable oxidation conditions (450 °C) and subsequent addition of the silicon resin through a physical coating method [70]. In this case, a compacting pressure of 1,200 MPa was used. Similar results are reported in Zhou et al. [85] for Fe-6.5 wt.% Si powder coated with 10 wt.% Ni_0.5_Zn_0.5_Fe_2_O_4_ nano-powder through spark plasma sintering. The maximum magnetic permeability of 855 was measured by Marinca et al. [68], producing a Fe/Fe_2_O_3_ composite powder through ball milling and reactive sintering (1100 °C). Slightly lower results are obtained in Yan et al. [80] through a similar technique using Fe-Si ferromagnetic powders. As for the mechanical properties, compressive strength of 244 MPa was achieved by Zhou et al. [85] whereas a TRS of 182.5 MPa is reported by Füzer et al. [84] with the adoption of Fe-Si with ferrite nano-fibers. Wang et al. [86] report a compressive strength of approximately 500 MPa, when a pure iron powder with a particles size of 20–40 μm was mixed with nano Ni-Zn ferrites powder using an agate mortar and spark plasma sintered process.

#### Different Production Methods of Ferrite-Coated SMCs

Ferrite coatings are suitable for different production processes, originating different properties and involving both simple and complex steps. The starting point is usually a ferromagnetic powder. The ferrite coating systems are based on different ferromagnetic powders: pure iron powder [68,69,87], Fe-Si powder [74,76,77,80,84,85,88], and Fe-Ni powder as Permalloy [89]. Various production processes can be used to coat ferromagnetic powder using ferrites. Most processes are based on ball milling and spark plasma sintering, as in [74,76,77,80,86]. Impact milling was also tested with promising results [69]. Other processes consist in surface oxidation [70,72,75]. In particular, Li et al. [72] performed in situ oxidation by mixing iron powder and water into a crucible and then putting it into a furnace at 150–300 °C. In this case, below 250 °C, there was no coating formation, while at 250 °C, the layer appeared very thin and brittle. The annealing process is often necessary after the compaction to increase the coating properties.

On the other hand, temperatures higher than 570 °C dramatically decrease the magnetite content increasing the eddy currents. In the work of Qian et al. [73], in situ surface oxidation was applied, along with hydrogen reduction, where the iron powder and deionized water were put into an autoclave reactor and then placed in a muffle furnace. The reduction steps are obtained by placing the previously oxidized powder in a tube furnace.

Other methods are based on sol-gel techniques, as in Lauda et al. [79]: the coating of the Fe-Si particles was carried out immediately after the gel creation and the auto-combustion process. The sol-gels methods are adopted because they show high versatility in producing ferrite layers [90,91,92,93]. In particular, Kumar et al. [94] used a natural solution Aloe Vera based to realize the coating. Other processes can be performed to produce ferrite coating systems for SMCs: the microwave treatment [82], coprecipitation [81] or using acetone [95].

Another method consists of alkaline bluing [83] by stirring the Fe powders with sodium hydroxide, sodium nitrate and sodium nitrite.

Füzer et al. [84] carried out coatings on Fe-Si spherical particles by ferrite nano-fibers Ni-Zn-Fe_2_O_4_ and by both ferrite nano-fibers Ni-Zn-Fe_2_O_4_ and phenolic boron-modified resin. The Ni_0.2_Zn_0.8_Fe_2_O_4_ ferrite nano-powder was obtained by ultrasonic breakage of ferrite polycrystalline nano-fibers prepared by needleless electrospinning methods. This technique allows obtaining medium-high mechanical properties.

**Figure 4:**
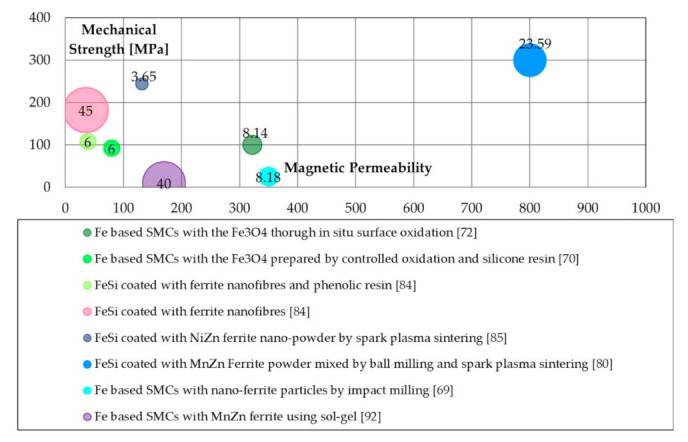
Magnetic (maximum permeability), mechanical (TRS [MPa]), and energetic properties (total iron losses 1 T@50 Hz [W/kg]) of ferrite coating processes.

Figure 4 reports the magnetic vs. mechanical and energetic properties of ferrite coated SMCs. The best mechanical properties of 300 MPa were noticed for ball milling and SPS, along with high magnetic permeability. Instead, low iron losses were obtained with nano-powder Ni-Zn coating and SPS.

### 3.5. Aluminum Alloys and Alumina

Aluminum is used to form inorganic insulating layers in SMCs. It can directly be part of the insulation, in the form of Al_2_O_3_ [96,97], or can be added to the ferrous powder to enhance its properties as alumina or aluminum based powder [98,99,100,101,102].

To produce an alumina coating directly on ferromagnetic particles, in [97], aluminum nitrate was used. The production of Al_2_O_3_ was carried out via one-pot synthesis in a reactor; the compaction was then performed by a hydraulic press applying 1200 MPa compacting pressure. However, the lubricant adopted during the compaction is less significant if the aluminum nitrate content increases.

Aluminum nitrate affects iron losses: as aluminum nitrate concentration increases, the loss amount decreases. Conversely, an increase in aluminum nitrate worsens the formability during compaction. Peng et al. [99] adopted nanoparticles of Al_2_O_3_ on Fe particles, coating them with silicone. Al_2_O_3_ nanoparticles are mixed with iron because of their high melting point, thermal stability, and electrical resistivity. Al_2_O_3_ addition provides good thermal resistance, allowing higher curing temperatures. Permeability is limited to 120: such a value is not adequate for electrical machines applications. Energetic performances of 6.86 W/kg (1 T @50 Hz) were noticed in SMCs hydrothermally coated with lithium aluminum oxide [103]. According to the literature [100], the maximum magnetic permeability for aluminum alloy admixed SMCs is 510 using an Al-Cu-Si-Mg alloy, with a TRS of 68.22 MPa.

#### Different Production Methods of Alumina and Aluminum Alloys Coated SMCs

Alumina and aluminum alloys can be used as a coating for various types of powders [104]. The sol-gel method finds application to produce alumina coatings [98,105]. Ball milling and spark plasma sintering may be used to cover ferromagnetic particles (Fe-Si-Al). In [106], Luo et al. adopted an insulation layer made in Al_2_O_3_/MnO_2_ for insulating Fe-Si-Al particles utilizing spark plasma sintering SPS technique. Conversely, the Fe_2_O_3_ addition along with Al_2_O_3_ is possible through hydrolysis precipitations [107].

It is also possible to coat the Fe-Si-Al based powders by nitridation or oxidation processes [96]. In this case, high purity N_2_ was provided to prepare low oxygen and high nitrogen atmosphere at 1100 °C to produce homogeneous Al_2_O_3_ and AlN insulating layers on the surface of Fe-Si-Al powders.

Several research activities were conducted by adopting Al alloys [100,101,102,108,109].

**Figure 5:**
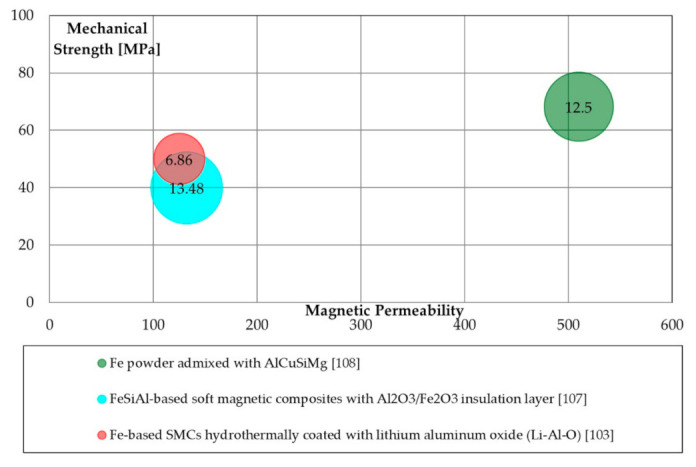
Magnetic (maximum permeability), mechanical (TRS [MPa]), and energetic properties (total iron losses 1 T@50 Hz [W/kg]) of alumina and aluminum alloys coating processes.

Figure 5 reports the magnetic vs. mechanical and energetic properties of Al and Al_2_O_3_ coated SMCs. Mechanical properties remain lower than 100 MPa while the iron losses are, in general, high.

### 3.6. Titanium

Titanium layers show exciting results, especially in terms of iron losses (about 3 W/kg at 1 T @50 Hz). Conversely, mechanical values at the moment are not primarily studied, causing a lack in the experimental data, mainly due to the amorphous spherical powders adopted (Fe-Si-B-C-Cr) [110,111]. The magnetic permeability is reported to be below 100. Sol-gel methods are commonly used [112] to obtain Ti layers.

### 3.7. Other Inorganic Layers

Various coatings are possible for SMCs as a function of shapes and material of both layer or ferromagnetic powders. For instance, in [113], fiber-based soft magnetic composites (FSMCs) were prepared using Fe fibers coated with a thin polymer layer. By the adoption of this technique, a very high magnetic permeability of about 900 is obtained.

Even Fe-Co may be used to produce SMCs [114,115], obtaining variable properties as a function of the production process. Fe-Co/Co-Fe_2_O_4_ micron-nano composites fabricated by controlled oxidation of micron-sized Fe-Co particles [114] providing iron losses values of about 6.5 W/kg at 1 T @50 Hz. On the other hand, magnetic permeability is around 70, while mechanical values are not reported in the literature. Weidenfeller et al. [115] produced SMCs using a spherical, gas atomized Fe-Co-V powder and wax.

Other researchers proposed a microwave technique to cover pure Fe microparticles (ASC 100.29) using MgO nanoparticles [116]. The produced SMCs showed good mechanical properties: TRS was 117 MPa, but the permeability remained limited.

Commercial gas-atomized Fe-6.5 wt.% Si (45 µm) powders and ZrO_2_ powders (30 nm) were mechanically milled by a ball mill [117] to realize a zirconia-based layer. In this case, the magnetic permeability and the iron losses resulted relatively low (5 W/kg at 1 T @50 Hz of iron losses, 230 of permeability).

Another example is given by Liu et al. [118] and involves HNO_3_ oxidation. The obtained coating is used for Fe-Si-Al powders. In this case, applied compacting pressures were about 1900 MPa. Fe-Si-Al powder presented in [119] were precoated by Ni through cold spray (CS) and high-velocity oxygen fuel (HVOF). Ni/Fe-Si-Al soft magnetic composite was obtained by hydrothermal hydrogen reduction process, providing low coercivity.

The spraying technique is a novel method for SMCs production. For this reason, it does not appear easy to find a complete characterization of the obtained SMCs. Moreover, some drawbacks are represented by the dispersion of large quantities of Fe-Si-Al during the process and the deformation and elongation of particles along the direction perpendicular to the deposition.

Hybrid SMCs are proposed as the mixture of different fractions of several ferromagnetic powders, kept together by a mixture of different binders [120]. For instance, Somaloy^®^ powder was mixed with a selected weight fraction of Vitrovac amorphous powder by Hegedus et al. [121]. Iron losses, in this case, resulted in about 3.5 W/kg (1 T @50 Hz). Conversely, a similar process was adopted in [122] by Perigo et al. by mixing pure iron and amorphous Fe-Si-B-C powders using two binders (wax and silicon resin), finding limited magnetic properties.

**Figure 6:**
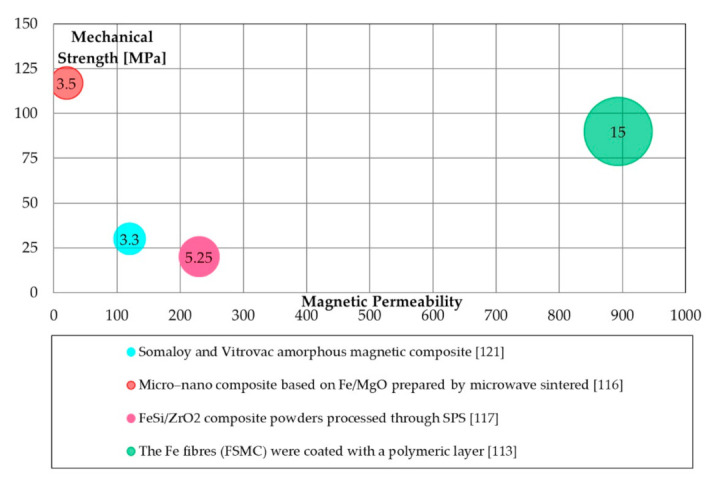
Magnetic (maximum permeability), mechanical (TRS [MPa]), and energetic properties (total iron losses 1 T@50 Hz [W/kg]) of other inorganic coating processes.

Figure 6 shows the magnetic, mechanical, and energetic properties of inorganic coated SMCs based on Mg, Zr, fibers and amorphous systems. Despite the very high permeability of 900, Fe fibers coated with a thin polymer layer showed high iron losses. On the other hand, low iron losses are obtained with MgO and Vitrovac coating, while magnetic permeability remains very low. The maximum mechanical properties measured were slightly higher than 100 MPa.

### 3.8. Inorganic Coatings: Properties Overview

All the inorganic coatings presented in the previous sections are characterized by pros and cons. In particular, Table 1 highlights the advantages and disadvantages of each coating system, focusing on the technologies adopted to realize it.

## 4. Organic Coating Systems

This section reports the organic coating systems commonly adopted in SMCs. Mainly, epoxy resins, phenolic resins, silicon resins, and thermoplastic resins systems were shown.

### 4.1. Epoxy Resins

Epoxy resins are often used in both inorganic and organic coating systems as mechanical binders. Nevertheless, organic coatings represent the primary materials forming the insulating layer. Epoxy resins additions affect the maximum operating temperature due to the low thermal resistance compared to inorganic layers.

Organic SMCs require a curing treatment in the range of 100–200 °C to increase mechanical properties attended and energetic properties. Also, curing treatment at room temperature is possible. In general, resins play an essential role as a lubricant, therefore avoiding lubricant addition into the mixture. They are also used in the heated die during compaction [123]. Various ferromagnetic powders can be coated with epoxy resin [124,125]. Particles size [20], resin content [126], and molding pressures [127] strongly impact the SMCs performances. In Pošković et al. [126], the minimum epoxy content weight was strongly reduced to 0.05 wt.%. Compacting pressure of 1,200 MPa was adopted by Shokrollahi et al. in [123].

The lowest iron losses were achieved in [124], 6.1 W/kg (1 T @50 Hz), using an epoxy resin to cover the amorphous powder Fe_73.5_Cu_1_Nb_3_Si_13.5_B_9_.

TRS of about 120 MPa were reported in [123,127], while the maximum magnetic permeability reached was 570 [123,126].

**Figure 7:**
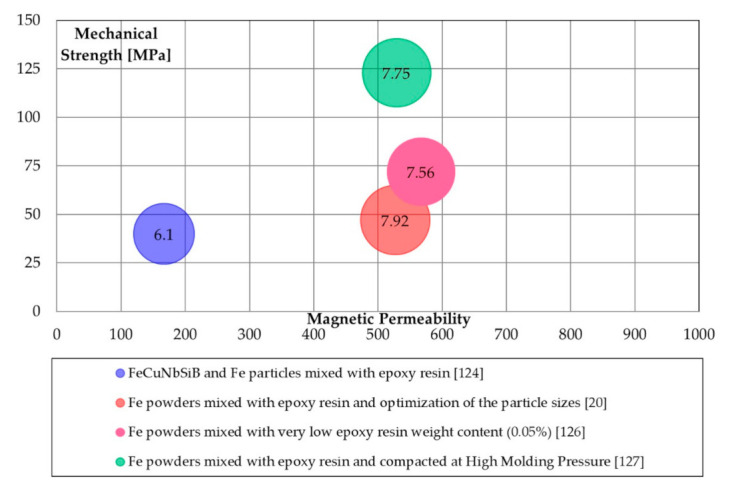
Magnetic (maximum permeability), mechanical (TRS [MPa]), and energetic properties (total iron losses 1 T@50 Hz [W/kg]) of epoxy coating processes.

Figure 7 illustrates the magnetic, mechanical, and energetic properties of epoxy resins coated SMCs. The iron losses resulted slightly higher in respect to the losses obtained with some inorganic coatings, while the average permeability observed in literature is almost 500.

### 4.2. Phenolic Resins

Phenolic resins are largely adopted in organic SMCs. The phenolic systems allow operating at temperatures higher than for epoxy resins (up to 300 °C). Furthermore, some phenolic resins are supplied in the form of powders, such as Novolac, simplifying their use [128,129]. Different types of ferromagnetic powders can be coated [19,128,130,131] and the production process combines metallurgical and polymeric processes [130,132]. Several phenolic resins commercially available were considered in [133]. The resin type, the content and the applied compacting pressure strongly affect the final densities and the energetic and magnetic properties [127,129,134,135].

Taghvaei et al. [129] noticed iron losses at 8.37 W/kg (1 T @50 Hz) adopting phenolic resin IP502 with particle size <63 µm containing a Novolac phenolic resin and hexamine as curing agent. Similar results (8.02 W/kg) were obtained by Ferraris et al. [127], adopting a Novolac phenolic resin and hexamine as the curing agent. The maximum magnetic permeability of 560 is reported by Kollar et al. [131], then adopted phenol-formaldehyde resin (Bakelite ATM) and acetone.

**Figure 8:**
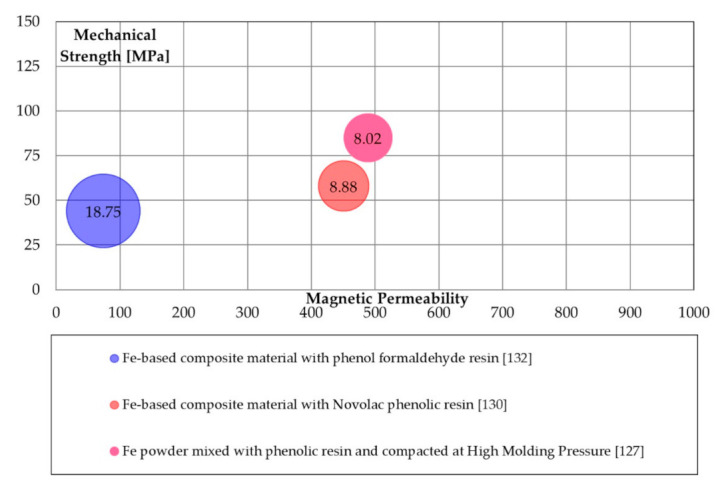
Magnetic (maximum permeability), mechanical (TRS [MPa]), and energetic properties (total iron losses 1 T@50 Hz [W/kg]) of epoxy coating processes.

Figure 8, the magnetic, mechanical, and energetic properties of phenolic resins coated SMCs are reported. The adoption of phenolic resin gave similar results as epoxy resins; however, a slight decrease in magnetic permeability was observed.

### 4.3. Silicon Resins

Silicon resins find applications with different ferromagnetic powders, but their peculiarity is the capability to operate up to 500 °C, as noticed for the silicone polymer Dublisil 20 (Dreve–Dentamid GMBH) in [133] and RTV (room-temperature-vulcanizing) silicone adhesive [136]. On the other hand, their drawbacks are the very low mechanical performances: the reported TRS (Transverse Rupture Strength) value in [136] (silicone adhesive) was 16 MPa, while in [137] a technical silicone was used, obtaining 1.18 MPa.

### 4.4. Thermoplastics

The production process is based on injection molding, as extensively discussed in [138]. Mainly, the matrix is polyamide 6 (PA6), thanks to its low viscosity and affordability [139]. Even polypropylene and rubber matrix can be used to prepare SMCs materials with thermoplastic polymers [140,141]. The main drawbacks are related to the low operating temperatures, low permeability, and a TRS of 16.5 MPa [141].

### 4.5. Other Organic Layers

Despite epoxy and phenolic resins being broadly adopted, other polymer materials can be adopted. In [142], for instance, there is the commercial diallyl-phthalate resin, which is filled with glass fibers and mixed with iron powder ASC 100.29. In this context, a mechanical TRS of about 39.87 MPa was measured, while magnetic and energetic properties, at the moment, are still not provided.

### 4.6. Organic Coatings: Properties Overview

All the organic coatings presented in the previous sections are characterized by pros and cons. In particular, Table 2 highlights the advantages and disadvantages of each coating system, focusing on the technologies adopted to realize it.

## 5. Conclusions

The present work explores a variety of processes and binders to produce SMCs. Given the multiple possible different uses in the electromagnetic field, identifying a single optimal product is practically impossible due to different reasons. Firstly, materials are often studied to meet the requirements of a specific application so that, for example, a composite used in a rotating electrical machine shows low performances in an RF inductor core and vice versa. Furthermore, comparing the SMCs outlined in different works is tricky due to a lack of uniformity in the test routines, measurement units, and data presentation. The use of non-SI measurement units and the partial absence of characterization data could be avoided, for example, by further standardization of the test routines.

Currently, SMCs still involve a tradeoff between different properties, limiting their wide use in new projects. Research activities are focused on investigating whether, when, and how these materials could play an important role in electrical machines, electromagnetic sensors, and RF devices.

## Figures and Tables

**Table 1:** Advantages and disadvantages in adoption of various inorganic coatings.

Coating Types	Technologies	Advantages	Disadvantages
Silica (Figure 1)	Epoxy-modified silicon resins	Easy processing; Good iron losses.	Poor mechanical properties; low magnetic permeability;
	Sol-gel	Good magnetic permeability; Good iron losses;	Low to adequate mechanical properties.
	SPS	High magnetic permeability.	Low mechanical properties.
Silicon (Figure 2)	Mixing, Milling	Easy processing. Adequate iron losses.	Low to adequate mechanical properties; low magnetic permeability;
	Sintered	Very high magnetic permeability; very high mechanical properties.	high iron losses; Expensive powder supply.
	SLM	Very high magnetic permeability; very high mechanical properties.	Expensive powder supply.
Phosphate-based (Figure 3)	Epoxy-modified resins	Easy processing; adequate iron losses.	Poor mechanical properties; low magnetic permeability;
	Insulation	Good magnetic permeability; high mechanical properties; good iron losses.	mold at 150 °C.
	Somaloy family	Good magnetic permeability; good iron losses.	Low to adequate mechanical properties.
Ferrites (Figure 4)	In situ surface oxidation	Adequate iron losses.	Adequate magnetic permeability.
	Epoxy-modified phenolic and silicone resins	Good iron losses.	Low magnetic permeability.
	Nanofibres	High mechanical properties.	Low magnetic permeability; high iron losses.
	SPS	Low iron losses; High mechanical properties.	Low magnetic permeability.
	Milling	Adequate iron losses.	Low mechanical properties.
	Sol-gel	-	Poor mechanical properties; low magnetic permeability; high iron losses.
Aluminum alloys and Alumina (Figure 5)	Admixed	Goog magnetic permeability.	High iron losses; low to adequate mechanical properties.
	Hydrothermal and hydrolysis	Good iron losses.	Low mechanical properties; low magnetic permeability.
Others (Figure 6)	Amorphous and ZrO by SPS	Low iron losses;	Poor mechanical properties; low magnetic permeability.
	MgO by Microwave	Low iron losses; Adequate mechanical properties.	Poor magnetic permeability.
	Polymeric layer coating	High magnetic permeability; Adequate mechanical properties.	High iron losses.

**Table 2:** Advantages and disadvantages in the adoption of various organic coatings.

Coating Types	Technologies	Advantages	Disadvantages
Epoxy Resins (Figure 7)	Mixing	Easy processing; good magnetic permeability; adequate iron losses.	Low to adequate mechanical properties.
	High molding Pressure	Good magnetic permeability; adequate iron losses; adequate mechanical properties.	High pressure level.
Phenolic resins (Figure 8)	Mixing	Easy processing; good magnetic permeability; adequate iron losses.	Low mechanical properties.
	High molding Pressure	Good magnetic permeability; adequate iron losses.	High pressure level.

## Data Availability

Data sharing is not applicable for this article.
